# Precise single column resection and reconstruction with femoral head plus total hip replacement for primary malignant peri-acetabulum tumors

**DOI:** 10.1038/s41598-024-52019-1

**Published:** 2024-02-10

**Authors:** Yongkun Yang, Yuan Li, Weifeng Liu, Xiaohui Niu

**Affiliations:** Department of Orthopedic Oncology Surgery, Beijing Ji Shui Tan Hospital, Peking University, Beijing, People’s Republic of China

**Keywords:** Oncology, Surgical oncology

## Abstract

To evaluate whether single acetabular column can be reserved and the effect of reconstruction with femoral head plus total hip replacement (THR) for primary malignant peri-acetabulum tumors. From 2007 to 2015, nineteen patients with primary malignant peri-acetabulum tumors were enrolled. All cases underwent single column resection with clear surgical margins. Ten of the 19 tumor’s resections were assisted by computer navigation. Femoral heads were applied to reconstruct anterior or posterior column defects; THR was used for joint reconstruction. The surgical safety, oncologic outcome and prosthesis survivorship and function were evaluated by regular follow-up. The average follow-up period was 65.9 months. Surgical margins contained wide resection in 12 cases and marginal resection in 7 cases. One patient with Ewing's sarcoma died 14 months postoperative due to lung metastasis. One case with chondrosarcoma had recurrence. One prosthesis was removed due to infection. The average MusculoSkeletal Tumor Society (MSTS) function score was 83.7%. Due to the relative small number of cases, there was no significant difference in the recurrence rate and prosthesis failure rate between the navigation group and non-navigation group. Single column resection and reconstruction with femoral head autograft plus THR is an effective, safe method with less complication rate and better functional outcome for patients with peri-acetabular tumors.

## Introduction

The incidence of pelvic tumor is low which accounts for approximate 4% of all bone tumors^1^. Due to the complexity of anatomical structure and important organs, the surgical treatment of pelvic tumor is difficult, therefore the intraoperative risk and recurrence rate is high^1,2^. More complications were found postoperatively^2–5^. With the improvement of oncological concepts and surgical techniques, more limb salvage surgeries were performed^6,7^. Therefore, more attention is paid to functional reconstruction and the control of complications^6,7^. According to Enneking and Dunham pelvic tumor classification^8^, tumor resection can usually lead to uncompleted and dysfunctional hip joint. Therefore, effective functional reconstruction is necessary but difficult.

There are several reconstruction methods for acetabulum such as inactivation and replantation^9,10^, saddle prosthesis^11,12^, semi pelvic prosthesis^13,14^, massive allograft^15,16^, or arthrodesis^17^. The replantation and pelvic prosthesis were relative common but brought significant complications such as infection, nonunion and fracture with unsatisfactory function^18^. For malignant pelvic tumors which involved single column, extensive resection can significantly reduce recurrence rate^19^. But the postoperative complications such as infection and delayed healing affected the postoperative functional recovery^19^.

Some special malignant pelvic tumor may only affect single column of acetabulum. To the best of our knowledge, there has been no special report on surgical treatment of this kind of tumors. Therefore, we focused on the operability, effect and safety of single column resection and reconstruction on malignant peri-acetabulum tumor. How to decrease the recurrence rate and apply effective reconstruction to bring benefit at the same time is a difficult problem. In recent years, we performed study on the reconstruction of acetabular tumors by strict preoperative planning and precise single column resection with respect to the surgical safety, oncological outcome and prosthesis survivorship and function.

## Materials and methods

### General characteristic

This was a retrospective case series study. This study was approved by the Ethics Committee of Beijing Ji Shui Tan Hospital (approval no. K201809-32), and informed consent was obtained from all patients. This study adhered to the declaration of Helsinki. All methods were performed in accordance with relevant guidelines and regulations. All cases were from the musculoskeletal tumor database in our department. From 2007 to 2015, nineteen cases were included according to the inclusion criteria: tumors located in the anterior or posterior column of acetabulum (zone II), also involved obturator area (zone III); zone I and IV was not affected by tumor; pathological confirmed primary malignant tumor; weight-bearing or stability was limited and reconstruction was necessary; surgical resection of single column and THR was performed. There were 9 males and 10 females. Mean age was 47.2 (24–64) years. Pathological diagnosis included 16 cases of chondrosarcoma (tumor grade: nine grade 1, six grade 2 and one grade 3), one case of undifferentiated pleomorphic sarcoma (UPS), one cases of Ewing's sarcoma and one case of solitary plasmacytoma. The lesion sites included 17 cases of anterior column and 2 cases of posterior column (Table 1).

**Table 1:** Clinical characteristics and surgical results of patients.

Gender	Age	Pathologic diagnosis	Site	Surgical margin (osteotomy)	Complication	Tumor relapse	Prosthesis failures
Female	36	CS	Anterior	Wide	Infection	No	Yes
Male	33	CS	Anterior	Wide	No	No	NO
Male	30	ES	Anterior	Wide	No	Metastasis	NO
Female	57	CS	Anterior	Wide	No	No	NO
Male	64	CS	Anterior	Marginal	No	Recurrence	Yes
Male	24	CS	Anterior	Wide	Infection	No	NO
Female	54	CS	Anterior	Wide	No	No	NO
Male	54	CS	Anterior	Wide	No	No	NO
Male	51	CS	Anterior	Wide	Infection	No	NO
Female	48	CS	Anterior	Wide	No	No	NO
Female	38	CS	Anterior	Wide	No	No	NO
Male	49	CS	Anterior	Wide	No	No	NO
Female	55	CS	Posterior	Wide	No	No	NO
Female	55	CS	Posterior	Wide	No	No	NO
Male	59	UPS	Anterior	Marginal	No	No	NO
Male	47	SP	Anterior	Wide	No	No	NO
Female	51	CS	Anterior	Wide	No	No	NO
Female	37	CS	Anterior	Wide	No	No	NO
Female	55	CS	Anterior	Marginal	No	No	NO

CS: chondrosarcoma; ES: Ewing’s sarcoma; UPS: undifferentiated pleomorphic sarcoma; SP: Solitary plasmacytoma.

### Preoperative examinations

**Figure 1:**
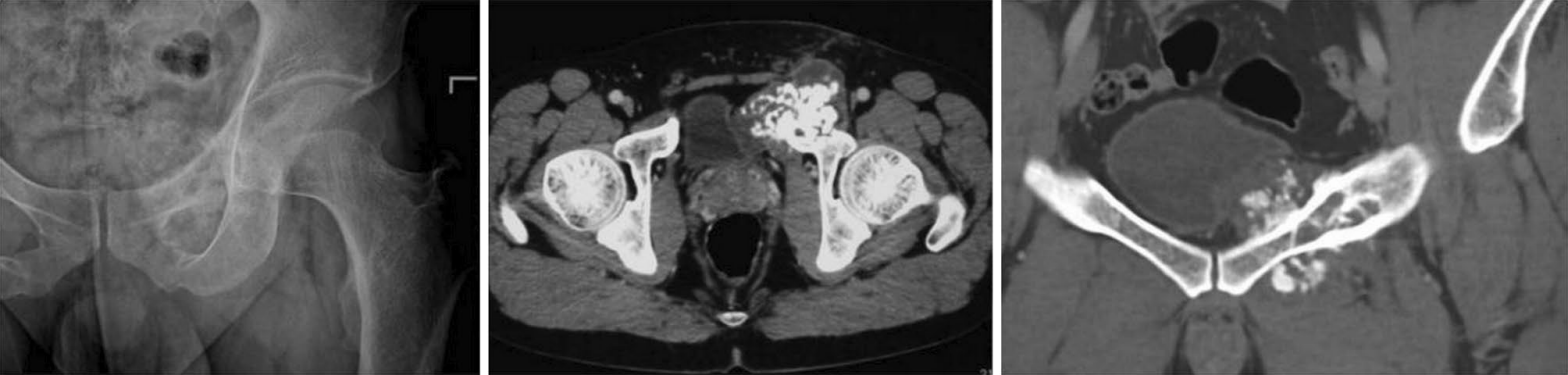
The preoperative radiography and CT of a 33 years male with chondrosarcoma of left anterior column of acetabulum.

All patients underwent clinical examination, laboratory examination, X ray, CT and MRI of pelvis and whole-body bone scan (Fig. 1). Pre-operative needle biopsy was performed for pathological examination. Definitive diagnosis was confirmed by postoperative pathology. The osteotomy line (wide margin) was designed as 1.5-cm margin from the edge of tumor.

### Surgical treatment

**Figure 2:**
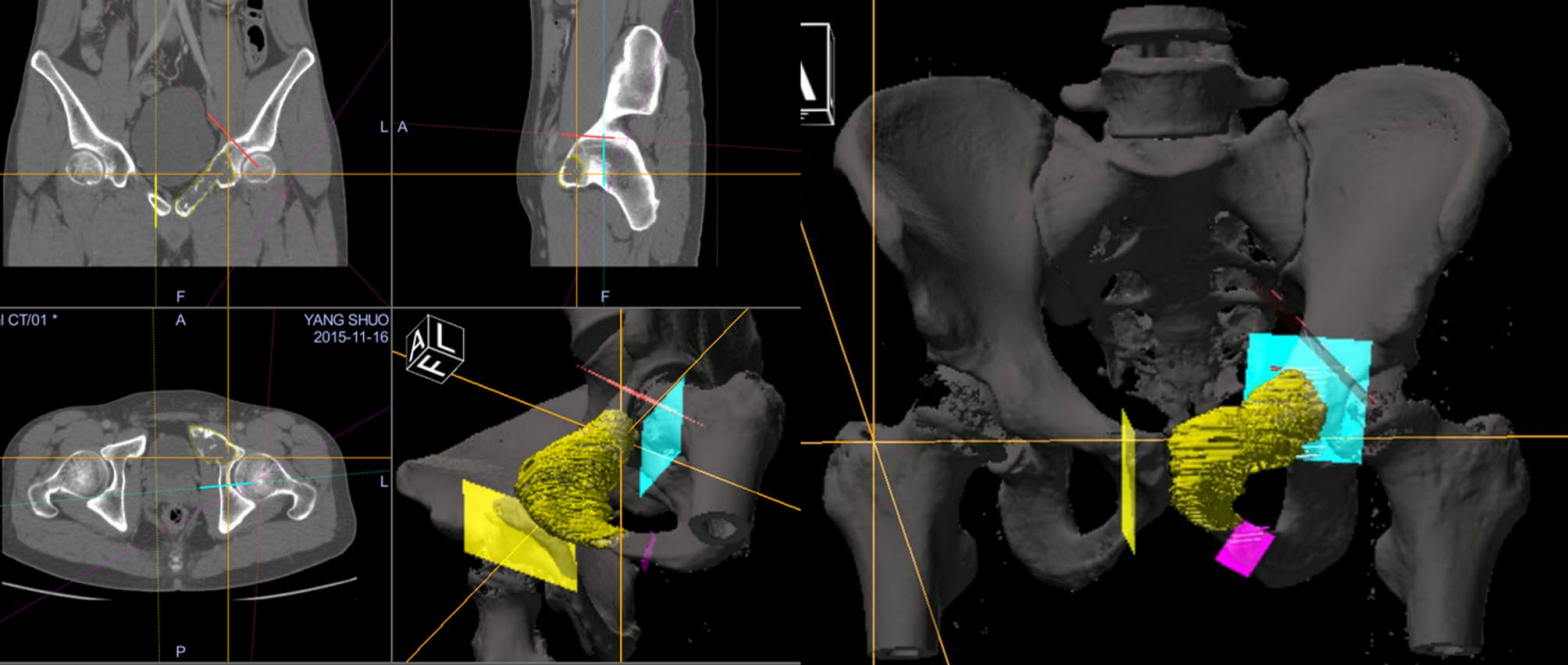
The tumor margin and resection plan was designed in navigation system. Tumor range was described as yellow area and the osteotomy planes were designed by virtual planes with different colors. Different cross sections and three-dimensional images showed the resection design (1.5-cm from the edge of the tumor). The anterior column of acetabulum could be resected safely and posterior could be reserved according to the preoperative plan.

**Figure 3:**
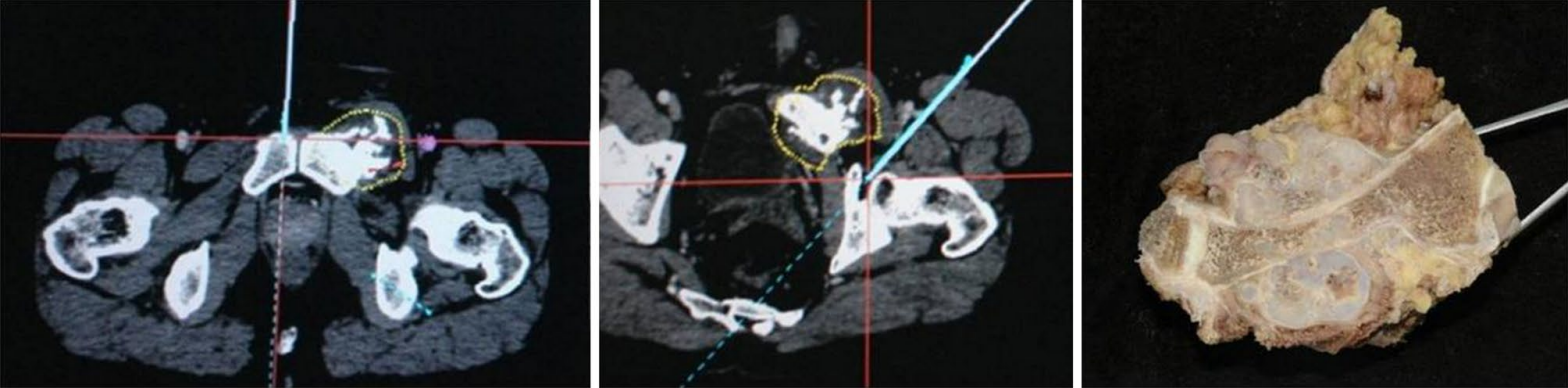
The precise tumor resection was performed under the direction of navigation system (intraoperative images of navigation). The postoperative specimen was cut and evaluated.

**Figure 4:**
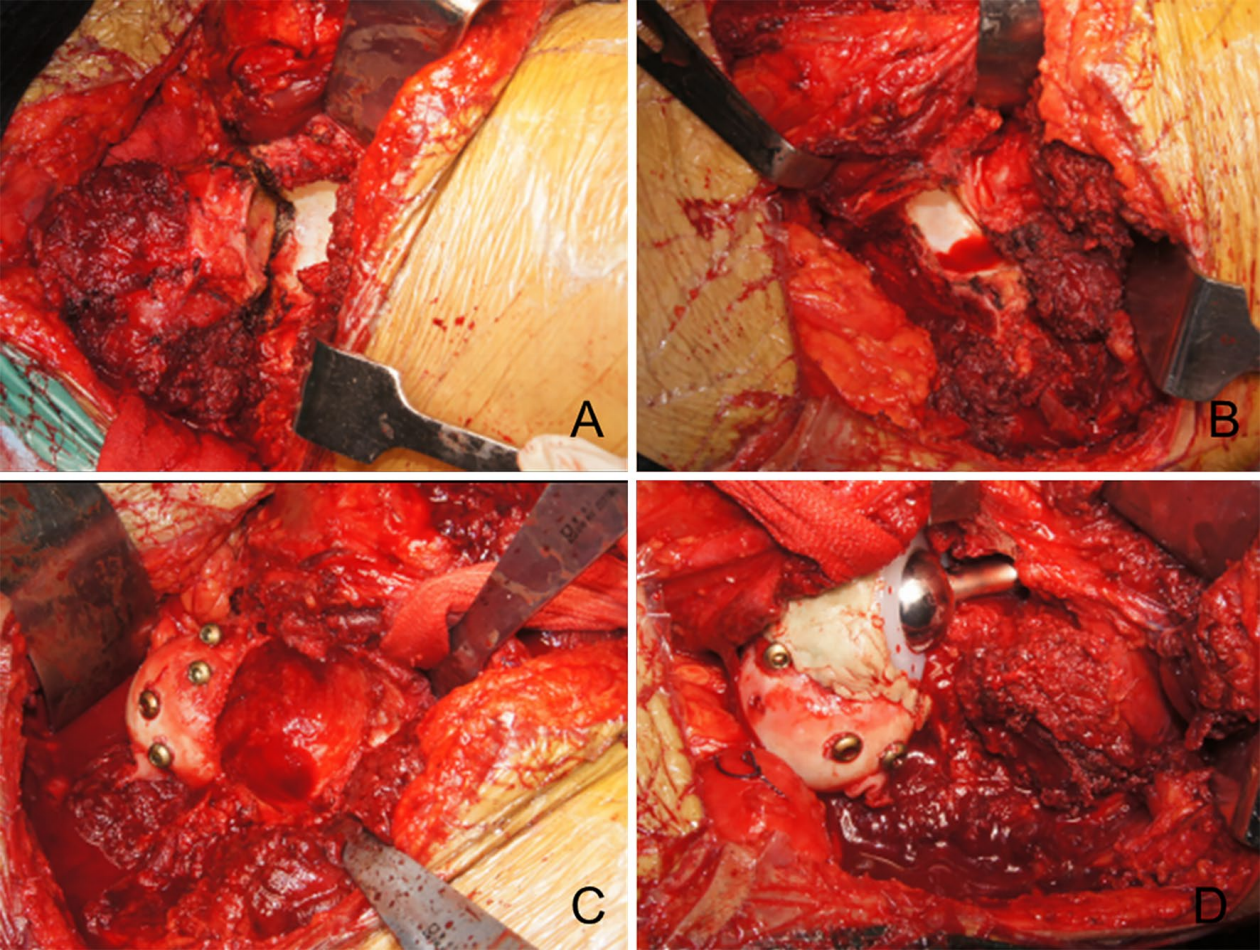
The precise single column resection and reconstruction with femoral head plus THR. (**A**) The bone resection line was marked with the direction of intraoperative navigation. (**B**) The precise single column resection was then performed. (**C**) The autologous ipsilateral femoral head was shaped and implanted in the acetabular defect. (**D**) The cemented THR was performed.

All cases underwent tumor resection performed by experienced senior surgeons. Computer navigation aided surgery was performed in 10 cases. CT and MRI images were fused in navigation system (Stryker Orthomap 3D Navigation System). The accurate three-dimensional model of tumor was showed in workstation of navigation and pre-operative resection planes and margins were designed (Fig. 2). Navigation aided tumor resection was performed intraoperative according to preoperative plan (Figs. 3, 4). After tumor resection, single column of acetabular had obvious bone defect. According to the shape and size of defect, autologous ipsilateral femoral head was shaped. And then it was implanted in the acetabular defect with surfaces of cancellous bone contacted A few long screws were used to fix it. The reconstructed acetabular was then shaped and cemented THR was performed. In addition, according to the site and stability of reconstruction, an acetabular reconstruction cage was used or not. The cage was applied in 12 cases. The postoperative specimen was cut and evaluated (Fig. 3).

### Followed up

**Figure 5:**
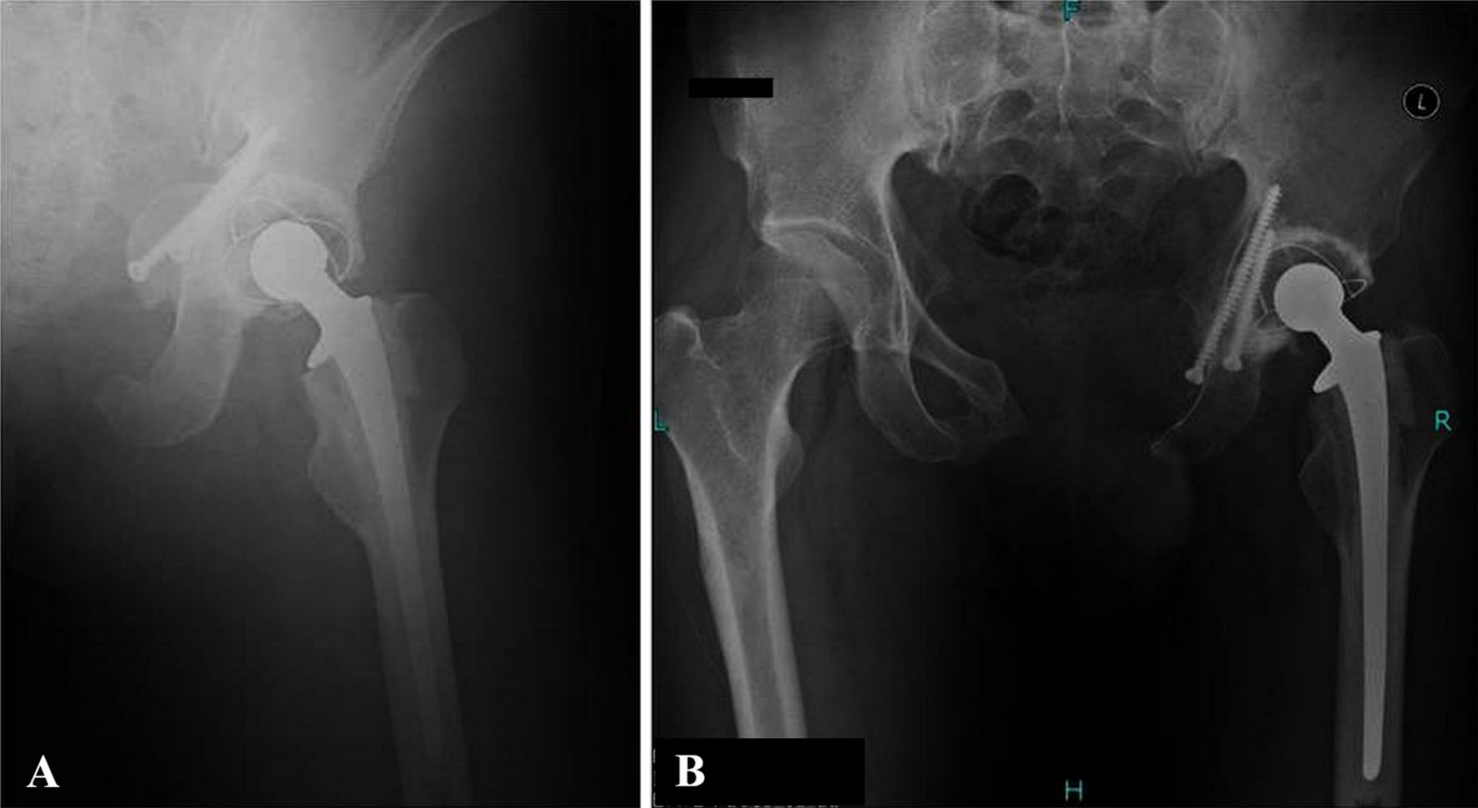
The radiography postoperative and 50 months postoperative.

Four cases (high grade chondrosarcoma, UPS, Ewing's sarcoma and solitary plasmacytoma) underwent post-operative chemotherapy. All patients were followed every 3 months postoperative (Fig. 5). The X ray and CT of the pelvis, chest CT and bone scan was performed. Postoperative limb function was evaluated with MSTS scoring system^20^.

### Statistical analysis

SPSS 20.0 software was used. The descriptive analysis, mean value t-test, chi square test, Fisher exact probability method and Kaplan Meier method was performed. P < 0.05 was considered as statistically significant.

## Result

### Surgical result

The average blood loss was 2800 (800–6000) ml and the average operation time was 333 (270–430) minutes. Tumor range and osteotomy line were designed before surgery. There were 12 wide resection, 7 marginal resection (low grade chondrosarcoma) and no intralesional resection. The planned osteotomy lines were obtained in all cases.

### Oncological results

The average follow-up period was 65.9 (median 65.5, range 12–149) months. One patient with Ewing's sarcoma died of lung metastasis 14 months postoperative. One patient with chondrosarcoma had recurrence 61 months postoperative and semi-pelvic amputation was performed. The recurrence rate in non-wide margin group and wide margin group was 14.3% (1/7) and 0 (0/12), respectively (p = 0.251). There was no significant difference in the recurrence rate between the navigation group 0 (0/10) and non-navigation group 11.1% (1/9) (p = 0.474).

### Prosthesis results

Two prosthesis failures occurred. One case was type IV failure (infection). The patient received arthrodesis 14 months postoperative due to deep infection. No other complication occurred 104 months post-operative. Another patient with type V failure (tumor recurrence) was the recurrent case mentioned above. There was no postoperative prosthesis dislocation and other prosthesis related complications. There was no significant difference in the prosthesis failure rate between the navigation group 0 (0/10) and non-navigation group 22.2% (2/9) (p = 0.211). The 5-year overall prosthesis survival rates were 90.9%. The average survival time was 102.8 (95% confidence interval, 86.1–120.0) months.

There were two minor wound infection: one case recovered after debridement and lavage; another case recovered after conservative treatment. The average MSTS score was 83.7% (25.1, range 17–29). The detailed average scores were as follows: 4.5 (4–5) in pain, 4.2 (2–5) in function, 4.3 (3–5) in emotional acceptance, 4.6 (all were 5 except 1 case needing crutch) in supports, 3.9 (3–5) in walking and 3.6 (2–5) in gait.

## Discussion

The wide resection of malignant pelvic tumor is more complex than limb tumor. Extensive exposure, long operation time and massive intraoperative bleeding increase the incidence of postoperative complications. The function of acetabulum structure is important, but large volume of the implant and the stress concentration cause complications^21,22^. The reconstruction of pelvic ring continuity and hip joint function is difficult. Long-term stable effect is also difficult to obtain^9–17^.

Because some special peri-acetabulum malignant tumor affect single acetabula column, we performed this study to evaluate whether partial acetabulum can be reserved and the effect of single column resection and reconstruction with femoral head plus THR for malignant pelvic tumors. The surgical safety, oncological outcome, prosthesis survivorship and function were analyzed.

Surgical resection of malignant peri-acetabulum tumor is difficult. The risk of operation and postoperative recurrence rate is high. De Paolis et al.^23^ reported 42 cases with long follow-up. There were 15 recurrences and 16 distant metastases. Deloin et al.^24^ reported 62 cases of pelvic chondrosarcoma. There were 18 local recurrences and 12 metastases. Surgical margin was significantly associated with recurrence. Poor margin, high tumor grade and acetabular involvement were risk factors of poor prognosis. Previous reports^25–34^ showed the recurrence rate of pelvic chondrosarcoma was 18–45% and surgical margin affected local recurrence.

Our study showed one local recurrence with chondrosarcoma after marginal resection. Therefore, the recurrence rate of chondrosarcoma was 6.3% (1/16). The recurrence rate was 14.3% (1/7) in non-wide cases and 0 (0/12) in wide cases. Therefore, the premise of good local control is safe surgical margin and the premise of obtaining a safe margin is accurate preoperative design and precise intraoperative performance. The excessive sacrifice of normal bone can obtain safe margin, but it also brings trouble in the reconstruction and increases perioperative complications.

A number of studies^12,35–37^ on saddle prosthesis showed high complication rates and unacceptable function. These included the destruction of iliac wing, prosthesis moving up, shortened limb length, prosthesis dislocation, wound infection, fracture and heterotopic ossification. Jansen et al.^36^ reported 17 cases of saddle prosthesis replacement with average MSTS score of 47%. Three patients couldn’t walk and 13 cases needed crutches. Fourteen cases had complications which contained 9 cases of infection and 2 cases of significant limb length discrepancy.

Semi-pelvic prosthesis can provide higher intensity and acceptable function, but the complications are unacceptable. The incidence rate was 75% and the most common was wound problems^38,39^. Therefore, it requires a careful consideration when selecting this kind of reconstruction. Abudu et al.^40^ reported 60% incidence of infection and dislocation, and 40% of the prosthesis were removed.

Reconstruction with massive allograft has high risk. Ozaki et al.^41^ reported 22 cases of allograft reconstruction and allograft was removed in 9 cases due to complications. Langlais et al.^15^ reported 13 cases with allograft reconstruction and 44% of them presented poor function. Yoshida et al.^42^ also reported very high incidence of complications in 19 cases.

Given high complication of saddle prosthesis, semi-pelvic prosthesis and massive allograft, some authors recommend arthrodesis^17^. But some problems also existed, such as activities limitation, limb length discrepancy, fusion failure and poor function^43–45^.

THR has certain advantages in the reconstruction of acetabular. Clayer et al.^46^ reported 29 cases with THR and only one case showed prosthesis loosening. Harrington et al.^47^ applied strengthening ring and screw fixation in THR in acetabular metastases. The postoperative results were good with no prosthesis loosening. Piya et al.^48^ reported 22 cases of metastases which received cement THR with screws fixation and cage was used in most cases. Only one hip dislocation and one superficial infection occured. Ho et al.^49^ reported THR with auxiliary of cement and screws. There were no prosthesis and cement loosening. Gill et al.^50^ suggested appling acetabular cup with wing in the reconstruction of acetabulum, the long-term result was similar with normal hip revision if no tumor recurrence.

In order to avoid complications of metal prosthesis, Puget et al.^51^ reported THR with autograft in the defect of acetabulum. The model had both advantages of prosthesis and autograft. It provided sufficient strength early term and biological healing of autograft long-term. The long-term result was better than pelvis prosthesis and allograft.

Our study showed two prosthesis failures and one was due to tumor recurrence. It suggested the main reason of prosthesis failure was tumor recurrence. Another failure was caused by deep infection. This failure reason is similar to conventional THR. There was no prosthesis dislocation and other prosthesis related complication. Our results showed high 5-year prosthesis survival rate. The mean postoperative MSTS score was 83.7% with acceptable daily activity. All patients could walk and take care of themselves.

Acetabular tumors require accurate resection and reconstruction. Our surgical resection and reconstruction depends on reliable preoperative design. Good local control can be obtained by accurate preoperative design and safe removal of tumor. Computer navigation has an advantage in our practice which contains accurate preoperative design and precise intraoperative real-time guide. The accurate resection and reconstruction can be achieved.

We retained single acetabulum to create condition for stable reconstruction. Autograft was applied to fill bone defect of acetabulum and bone fusion was achieved. Compared with artificial pelvis, saddle prosthesis or allograft bone, autologous biological reconstruction has obvious advantages. It can reduce the risk of infection, non-healing, fracture and prosthesis loosening. Three cases with infection were found in all 19 cases. Only one case underwent prosthesis removal and arthrodesis. The other two prostheses had good long-term results. Few complications and satisficed postoperative results were showed in our study. With the development of 3D printing technology and personalized reconstruction, we can perform comparative study between autologous bone and metal acetabular augment reconstruction in future.

There were some limitations in our study. First, it was not a prospective case control study. Second, the sample size was relatively small. The cases in our study were special that single column was involved by tumor and anterior/posterior column can be reserved. Thus, it’s difficult to find many cases suitable for this special operation. Selection bias may exist in our study and many peri-acetabular pelvic malignant tumors are not suitable for this surgical method. The very low recurrence rate and complications may be also related to selection bias. Third, the follow-up period was relative short and some patients were followed less than five years.

In conclusion, early results showed that single column resection and reconstruction with femoral head autograft plus THR is an effective, safe method with less complication rate and better functional outcome for patients with peri-acetabular tumors Partial acetabulum can be reserved through precise plan of resection and reconstruction. Additional studies comparing this method with alternatives and long-term results are required.

A shorter conference version of this paper was reported in CAOS 2018. The 18th Annual Meeting of the International Society for Computer Assisted Orthopaedic Surgery. This manuscript provides a richer data analysis and discussion of related content.

## Data Availability

The datasets used and/or analyzed during the current study available from the corresponding author on reasonable request.
